# The Influence of the Use of Whole Grain Flour from Sprouted Wheat Grain on the Rheological and Microstructural Properties of Dough and Bread

**DOI:** 10.1155/2021/7548759

**Published:** 2021-07-28

**Authors:** Natalia Naumenko, Irina Potoroko, Irina Kalinina, Rinat Fatkullin, Eva Ivanisova

**Affiliations:** ^1^South Ural State University (National Research University), 76 Lenin Avenue, Chelyabinsk 454080, Russia; ^2^Slovak University of Agriculture in Nitra, Tr. A. Hlinku 2, 949 76 Nitra, Slovakia

## Abstract

Whole wheat flour from sprouted wheat grain is a full-fledged raw ingredient containing essential amino acids, easily digestible sugars, and dietary fiber, with increased digestibility and enzymatic activity. The use of this raw material in the production of food products will contribute to the creation of products for a healthy diet of the population. This study is aimed at studying the possibility of using whole grain flour from sprouted wheat in the production of bread and its effect on the rheological and microstructural properties of dough and finished products. It was found that whole wheat flour from sprouted wheat grain had an even particle size and was characterized by a uniform distribution of particles over the size range (from 53 to 209 microns—61 ± 3%); large particles from 297 to 497 microns were present in an amount of no more than 10 ± 3%. The replacement of 20% refined flour with whole wheat flour from sprouted wheat grain resulted in better values of the farinograph quality index (200 ± 3 mm). The bread obtained according to this recipe had a high specific volume (4.21 ± 0.62 mL.g^−1^) and optimal rheological characteristics: total deformation13.7 ± 0.3 mm, plastic4.3 ± 0.3 mm, and elastic9.4 ± 0.3 mm. The study of the microstructure of dough and bread also confirmed the established dependencies. This percentage of replacement of refined flour with whole wheat flour from sprouted wheat grain can be recommended as the best for obtaining bread of good quality with high rheological characteristics.

## 1. Introduction

In recent years, sprouting of grain crops has been actively used to obtain new raw materials used in the manufacture of food products. This is due to an increase in their nutritional value and improved nutrient absorption [1]. A number of authors indicate that products from vegetable raw materials obtained using sprouted grain have a better taste, a softer consistency, and a pronounced sweet taste [2]. It is known that the germination process activates enzymes, thereby helping to increase the digestibility of the grain. During grain germination, the availability of reducing sugars and free amino acids, including lysine, increases [3] and stimulates the accumulation of gamma-aminobutyric acid [4], minerals [5], dietary fiber [6], and phenolic compounds and increases antioxidant activity [7].

The increase in enzymatic activity caused by germination can negatively affect the rheological properties of the dough and the quality of the finished bread, since the germination process reduces the baking properties of the flour. At the same time, this process is considered as a tool for improving the quality of food and increasing the functional potential of health promotion [6]. This is because starch becomes more digestible; there is an increase in the bioavailability of amino acids and, in addition, a large number of biologically active compounds.

Whole grain flour is rich in vitamins, minerals, and dietary fiber, as all the components of the wheat grain are completely absorbed into its composition, making it a raw ingredient of high nutritional value. However, the effect of wheat germination on the physico-chemical, rheological, and baking properties of flour and its microstructural changes has not been properly studied in the previous works, and it is of scientific interest.

The aim of the study was to evaluate the possibility of using whole grain flour from sprouted wheat in the production of bread and its effect on the rheological and microstructural properties of dough and finished products.

## 2. Materials and Methods

### 2.1. Materials

This study used a grain of soft spring white-grain wheat (*Triticum aestivum L.*), a variety of Lubava, harvested in 2014-2018, grown in the Ural region, Russia (grains nongerminated). The protein content was 12.5 ± 0.3 g/100 g in terms of humidity.

The ingredients used to make the bread were purchased at a market in the city of Chelyabinsk. Refined wheat flour (RF) (gluten 30.5%, ash content 0.55) was provided by the manufacturer OOO Soyuzpishcheprom, Chelyabinsk, Russia, and was used as the main raw material in the manufacture of bakery products.

Yeast and salt were purchased in the retail network of the city of Chelyabinsk (pressed baking yeast *Lux extra*, manufacturer of OOO SAF-NEVA, food salt of the highest grade, manufacturer of OOO Russol).

### 2.2. Production of Whole Wheat Flour from Sprouted Wheat Grain (WWF)

#### 2.2.1. Germination of Wheat Grain

To remove the contamination products and foreign substances, the wheat grain was prewashed in running water at 20 ± 2°C in a fivefold repetition. The grain (500 g) was soaked in water 22 ± 2°C for 6 hours until a humidity of 35% was reached. The samples were then placed in germination trays, which were located in a temperature- and humidity-controlled chamber (SHPZ seed germination cabinet, Russia). Germination was carried out at a temperature of 22 ± 2°C and a relative humidity of 95 ± 3%. The sprouted wheat was removed from the chamber after reaching the sprout size of 1.5-2.0 mm in more than 90% of the grains; the germination time was 14.6 ± 0.8 hours [8].

The sprouted wheat was dried (drying cabinet M 720, Binder, Germany) at a temperature of 35–40°C for 10 hours until the final moisture content of 8–14% was reached.

#### 2.2.2. Production of Whole Wheat Flour from Sprouted Wheat Grain

Whole wheat flour was obtained by milling the grain using a Perten 3100 laboratory mill, with a fixed speed of 20,000 revolutions per minute (Perten Instruments, Sweden), equipped with a 0.8 mm metal mesh; then, the flour was sifted through a 0.6 mm sieve [9]. The milling time was 180 seconds until the flour with stable particle sizes was obtained.

### 2.3. Evaluation of the Functional Properties of Sprouted Whole Wheat Flour

#### 2.3.1. Hagberg Falling Number (FN)

The value of the drop number (FN) was determined using the international method AACC 56-81.03 with the FN 1500 system (Perten Instruments, Sweden) with a flour sample size of 7 g (14% of humidity) in 25 ml of water.

#### 2.3.2. Distribution of the Average Particle Size of Flour Samples Was Carried Out by Laser Dynamic Light Scattering on the Microtrac S3500 Device (AASS 55-40.01, 2010)

*(1) Physico-Chemical Analysis of Whole Wheat Flour*. The moisture content was determined using the international method AACC 44-15.02. The protein content in whole wheat flour was calculated using the Kjeldahl micromethod [10]. The mass fraction of fat in whole wheat flour was determined by 30-25.01; the ash content according to AACC 08-12.01; the total amount of starch, AACC 76-13.01; and the index and gluten content, AACC 38-12.02 [10]; the dough was kneaded in accordance with the AACC 54-21.02 (2010) method on the Farinograph-AT (Brabender, Germany).

### 2.4. Rheological Properties of the Dough

For the use of whole wheat flour from sprouted wheat grain in the production of bread, mixtures of RF and WWF were prepared in the following proportions: (a) 90 : 10, (b) 90 : 20, and (c) 70 : 30.

The rheological properties of the RF and WWF dough were determined in accordance with the AACC 54-21.02 (2010) method on a Farinograph-AT (Brabender, Germany).

### 2.5. Scanning Electron Microscopy (SEM) of the Dough

The dough was prepared according to the method of Kim et al. [11], with some modifications, using Farinograph equipment. The dough samples were dried on freeze-drying equipment (Coolvacuum Technologies Lyomicron-55C, Spain). To obtain micrographs, a scanning electron microscope (JSM-7001F (JEOL), Japan) was used at 20 kV and a magnification of 1000x.

### 2.6. Test Laboratory Baking

The test laboratory baking was carried out using the international method AACC 10-10.03. The recipe of the obtained samples is presented in Table 1.

The amount of water was calculated based on the water absorption capacity of the flour at the rate of 650 E.F. (± 20) on the Brabender Farinograph equipment. The water temperature was 22 ± 2°C.

The dough was divided by hand into portions of 450 g, and fermentation was carried out in a controlled fermentation chamber (Atrepan 18/10, Italy), at 30 ± 1°C and a relative humidity of 80% for 90 minutes. Baking was carried out in a laboratory oven (Fin Bake II 5 D Digital, Slovenia) at 220°C for 20 minutes. The bread was cooled at room temperature and tested after 3 hours.

### 2.7. Bread Quality Assessment

The volume of bread was determined by the seed displacement according to the method AACC 10-05.01, 2010. The bread was weighed, and its specific volume was calculated. The moisture content of the bread crumb was determined according to AACCI 44-15.02, 2010. The deformation of the bread crumb was determined in accordance with the AACC 74-09.01, 2010 method using a texturometer (Structurometer ST-2, Russia). From the bread samples (4 hours after baking), slices up to 25 mm thick were cut, reducing the size of the slices to 25 mm long by 25 mm wide, and the crust layer was removed. For the analysis, an aluminum cylindrical probe P/20 (radius 20 mm) was used, and the following experimental parameters were used: preliminary testing of 1.0 mm s^−1^, test of 1.7 mm s^−1^, reverse stroke of 10.0 mm s^−1^, and compressive force of 40%.

### 2.8. Scanning Electron Microscopy (SEM) of Bread

The microstructure of dough and bakery products was studied using transmission electron microscopy (JSM-7001F (JEOL), magnification 1000x). The samples were predried on freeze-drying equipment (Coolvacuum Technologies Lyomicron-55C, Spain), then sputtered with platinum.

### 2.9. Statistical Processing of Results

The studies were conducted in a fivefold repetition. Grain germination, whole wheat flour production, and bread samples were carried out under the same conditions to ensure the accuracy of the results. The experimental data were processed on the basis of mathematical statistics methods using Microsoft Excel and MathCad. The obtained data are presented with a confidence factor of 0.95.

## 3. Results and Discussion

### 3.1. The Influence of the Germination Process on the Physical and Chemical Parameters of Whole Wheat Flour from Sprouted Wheat Grain

Previously, Olaerts et al. described that the germination process leads to a significant decrease in FN, which is associated with increased activity of the alpha-amylase enzyme in wheat grain [12]. Based on the literature [13], *α*-amylase is considered one of the main limiting factors for use in the production of bread flour from sprouted wheat grain; it is able to hydrolyze starch molecules, which leads to a sharp decrease in the viscosity of the dough, loss of shape stability of the products, and the production of sticky crumbs. In the process of making bread, different batches of flour with different FN values can be mixed to produce high-quality bakery products.

In this study, the change in the FN value was determined during the germination of wheat grain in order to control this process [14]. It was found experimentally that after the germination of the organs of the sprout and the beginning of the appearance of the root system, the values of the FN index reach a value of 75 ± 10 seconds (Table 2), which means that it is possible to obtain high-quality bread only with a partial replacement of RF with WWF.

According to [15], milling is one of the most important processes for processing sprouted grain. The production of fixed-size flour particles is the main milling criterion used in grain processing and determines the suitability of flour for further processing. In the production of flour, roller mills are widely used for this purpose, but hammer mills can also be used [15]. The processes that occur during germination affect the biochemical changes as well as the structural changes in the grain. As a result, the strength properties of the grains change, especially the hardness of the wheat grain and the energy spent on milling are reduced. Sprouted grains are not recommended to be ground by classical milling, since the high plasticity of the grains reduces the yield of flour and increases the ash content in the flour [16]. Ariyama and Khan (1990) studied the milling properties of sprouted and common wheat [17]. They found that the biologically active compounds in the grain are mainly located in the shell parts, so the production of flour by single milling for this type of grain raw material is most preferable.

Based on the literature [18], the process of preparing the sprouted grain for milling is simple, because the grain mass is cleaned before the germination process is carried out. For dry milling, the moisture content of the sprouted grain should be reduced to an optimal level (no higher than 14%). [9, 19] expressed that the results of the milling process also strongly depend on the type of mill used. In our work, we used a laboratory mill Perten 3100, with a fixed speed of 20,000 revolutions per minute. The resulting flour was sifted through a 0.6 mm sieve. The milling time was 180 seconds until the flour with stable particle sizes was obtained. To characterize the milling process, such characteristics as the particle size distribution and the weighted average particle size were used.

**Figure 1:**
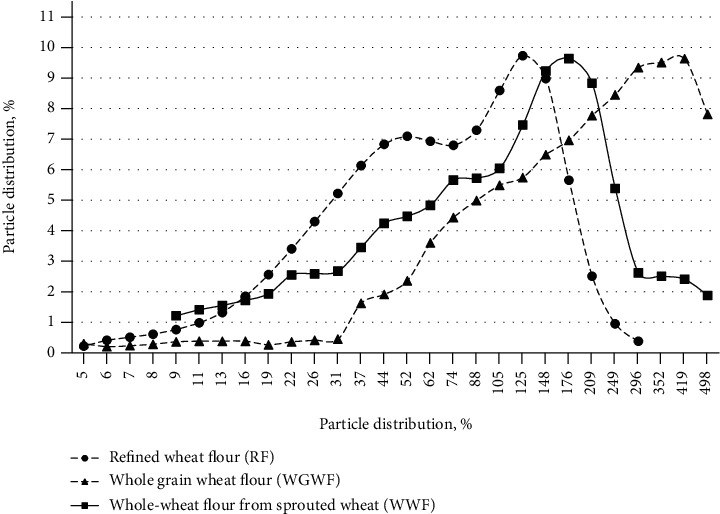
Profile of distributing size particles of refined wheat flour and whole wheat flour from sprouted and nonsprouted wheat grains.

The particle size distribution is a very important factor from a technological point of view, as it affects the properties of the flour and the subsequent processing steps [20]. The results showed that the crushed material obtained from whole grain wheat flour (WGWF) wheat grain was characterized mainly by a higher proportion of large particles (more than 209 microns) and a low proportion of small particles less than 52 microns—9 ± 3% (Figure 1).

A strong influence of the germination method on the particle size distribution was noted; the WWF sample was characterized by a uniform distribution of particles over the size range; large particles from 296 to 498 microns were not more than 10 ± 3%.

In all flour samples, the moisture content showed values close to 13.5%; the ash content did not change during germination. The slight increase in flour lipids during germination may be due to an increase in free lipids provided by the germination process [21]. As for the protein content relative to the WGWF sample, the WWF flour sample showed an increase of 0.7%. The values of the gluten index and the content of wet gluten in WWF flour differed significantly (*p* ≤ 0.05) from WGWF.

WGWF flour has the lowest gluten index and the lowest wet gluten content, which can be attributed to its low gluten quality and low protein content in nonsprouted wheat. The WWF sample has large gluten values. These findings are consistent with studies by Hadnadev et al., which reported similar results, where wheat most exposed to high temperatures and precipitation prior to harvest presented the highest values of wet gluten content [22]. The gluten index of the WWF sample is slightly higher than the WGWF. This change is consistent with research by Ichinose et al., who observed differences in gluten quality even without detecting changes in proteolytic activity [23].

The changes observed in the formation of the gluten structure after germination may be due to the formation of phenolic compounds capable of binding sulfides available for the formation of disulfide bridges. This is important for the formation of the structure of gluten, and protein oxidation and strengthening of gluten can also occur [19, 24].

### 3.2. Rheological Properties and Scanning Electron Microscopy of the Dough

Bread manufacturers do not want to get flour from wheat with a low FN, because products made from wheat flour with a low FN value have a sticky, crumbly crumb, and a low specific volume [25]. While wheat flour with high FN will also not produce good bread, there is a need to add additional malted raw materials or *α*-amylase tablets, in order to increase the enzymatic activity and increase the volume of bread. Therefore, in further studies, the addition of part of the flour from the sprouted wheat grain was carried out by partially replacing the refined flour. Mixtures of RF and WWF were prepared in the following proportions: (a) 90 : 10, (b) 90 : 20, and (c) 70 : 30. A dough sample obtained from 100% RF was used as a control. The effect of replacing refined flour with whole wheat flour from sprouted wheat grain on the rheological characteristics of the dough is presented in Table 3.

The control sample of the refined flour dough was characterized by high water absorption (61%) and fairly good stability (8.2 min), which is typical for wheat flour with a sufficiently strong gluten.

Replacing RF with WWF resulted in a significant (*p* ≤ 0.05) decrease in water absorption. Pasqualone et al. in their studies [26] point out that the use of raw materials rich in dietary fiber increases water absorption due to their considerable hygroscopicity. In this case, however, we must consider the WGWF and the WWF as a complex system, with high enzyme activity, affecting the farinograph quality indicator. According to Dojczew and Sobczyk, the decrease in water absorption may be mainly due to the depolymerization of proteins as a result of the intense activity of proteases in sprouted wheat [27]. A number of authors [28–30] of the previous studies noted that the activity of proteolytic enzymes increases during the germination. They (proteolytic enzymes) hydrolyze gluten and partially break down high-molecular-weight proteins into simple ones. These changes significantly affect the rheological properties of the dough, reducing its water absorption capacity.

**Figure 2:**
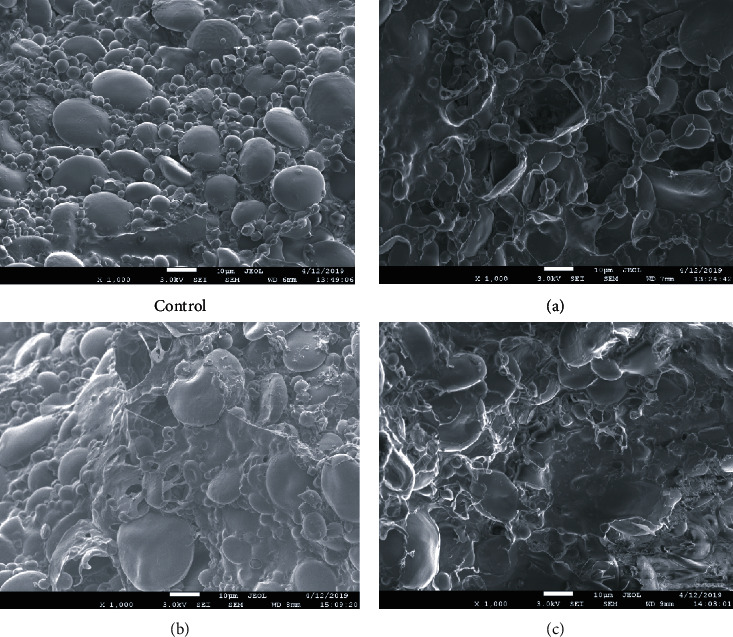
Images of wheat dough samples obtained using a scanning electron microscope (SEM): Control, a dough obtained from RF; a mixture of RF and WWF in the following proportions: (a) 90 : 10; (b) 90 : 20; (c) 70 : 30.

The dough development time increases slightly with the introduction of WWF (from 2.5 to 4.7 minutes); the dough stability increases before replacing RF with WWF in the amount of 20%, and when replacing 30%, it drops sharply to 4.4 minutes, indicating a weakening of the dough as a result of the increased action of *α*-amylase. It can also be noted that 30% of the replacement of RF with WWF led to a sharp decrease of the dough consistency and the farinograph quality indicators. A similar trend in the dough was demonstrated when soft wheat and durum wheat flour were mixed in equal portions [31]. This phenomenon may be due to differences in the interactions between the gluten proteins from RF and WWF; similar results were found for the proteins of soft wheat and gluten of durum wheat [32, 33]. This hypothesis must be investigated further before any definitive conclusions can be drawn. The replacement of 10 and 20% RF with WWF obtained the dough with the high stability and farinograph quality indicators, which agree with the data of Marti and his colleagues and may be associated with a nonsignificant destruction of the gluten matrix by enzymes and moderate action of *α*-amylase [34]. The microstructure of the dough samples under study is shown in Figure 2.

The structure of both the control and experimental dough samples (a, b, c) is characterized by the presence of a large number of oval-shaped particles with a size of 5 to 30 microns, which in their characteristics correspond to starch grains. The grain surface is smooth, without cracks, grooves, and pores [35]. It is possible to distinguish the presence of large, medium, and small starch grains. Starch granules are clearly distinguishable, have a spherical shape, are visualized on the surface, and have a similar structure.

In the dough samples (control and a), more fine- and medium-sized starch granules are visualized. Starch is present in the form of round or elliptical grains. Individual grains are slightly deformed; more often, this is observed in large starch grains. Individual starch grains have attached protein particles, which gives them an angularity. In samples (b) and (c), swollen, significantly increased in size large starch grains with a size of 20 to 30 microns predominate.

The protein matrix is developed in all samples somewhat differently. In the control sample, it is not sufficiently developed; only in rare cases, several grains of medium and fine starch are covered with a layer of protein (Figure 1 (control)). The structure is quite loose, as there are air cavities. In sample (a) (Figure 1(a)), in some cases, we can consider the structure of a discontinuous protein matrix, which has the form of processes connecting starch grains. The free globules of the protein are not visible.

In samples (b) and (c), the protein matrix is more clearly distinguishable, evenly distributed, and surrounds most of the starch grains, combining them. Numerous holes from the formed air bubbles are visualized on the surface of the protein matrix, which indirectly confirms a more intensive fermentation process of the dough.

### 3.3. Quality of Finished Products, Rheological Properties, and Scanning Electron Microscopy of Bread Crumb

**Figure 3:**
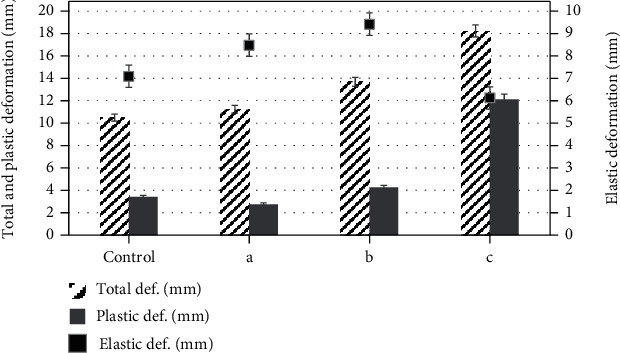
Deformation characteristics of bread crumb samples. The error bars represent the standard deviation of three separate measurements for five samples (*n* = 15). Sample designation: control, crumb of bread obtained from RF; (a) crumb of bread obtained from a mixture of RF and WWF in the proportions of 90 : 10; (b) crumb of bread obtained from a mixture of RF and WWF in the proportions of 90 : 20; (c) crumb of bread obtained from a mixture of RF and WWF in the proportions of 70 : 30.

The influence of WWF on the quality of bread was evaluated by the specific volume, humidity, and deformation characteristics of the crumb (Table 4 and Figure 3). The use of WWF (a, b) increased the specific volume of bread. The specific volume of bread with 10% WWF was about 7% higher than the control sample and with 20% by 15%, while the introduction of 30% of WWF, on the contrary, reduced this figure by 17.3%. The moisture content of all bread samples ranged from 40.2 to 41.6%; similar values were reported by Boita et al. [36].

The addition of WWF also had a positive effect on the deformation characteristics of the bread crumb. In samples (a) and (b), a slight increase in the total deformation index can be noted, while the elastic deformation also has high values, which indicates a high recovery capacity and a well-developed porous structure.

Several studies have shown [37, 38] that the production of hydrolytic enzymes during germination contributes to the improvement of crumb softness. In particular, *α*-amylase reduces the retrogradation of amylopectin and makes the crumb structure more elastic [39].

At the same time, an excessive amount of WWF, which is observed in the sample (c), leads to a significant increase in the plastic deformation index, which indicates the formation of a sticky, jammed crumb, and a deterioration in the quality of bread. This fact may be due to the weakening of gluten, which makes it difficult to retain gas in the dough and contributes to a denser crumb.

**Figure 4:**
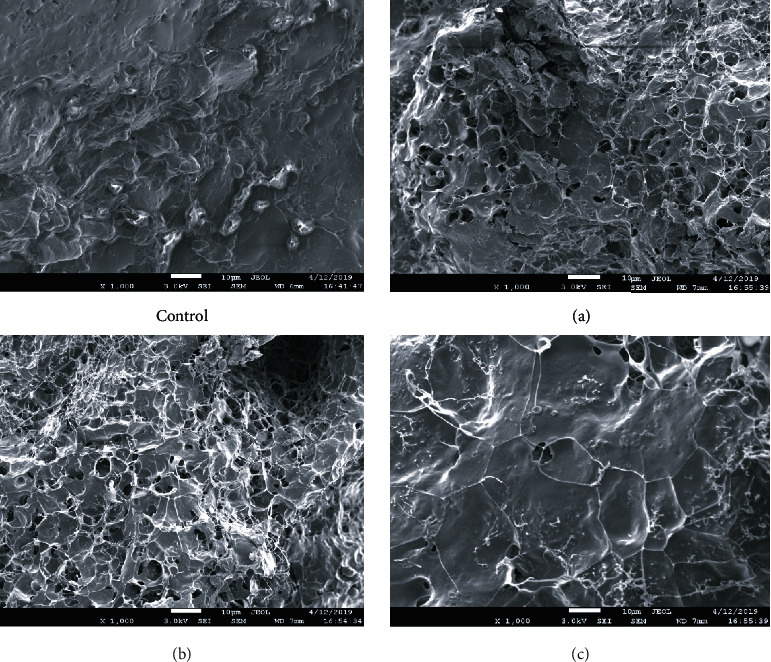
Images of bread crumb samples after 4 hours of storage obtained using a scanning electron microscope (SEM). Sample designation: control, crumb of bread obtained from RF; (a) crumb of bread obtained from a mixture of RF and WWF in the proportion of 90 : 10; (b) crumb of bread obtained from a mixture of RF and WWF in the proportion of 90 : 20; (c) crumb of bread obtained from a mixture of RF and WWF in the proportion of 70 : 30.

The crumb of bread after baking can be represented as a single structural system of starch, protein, and moisture molecules. The crumb structure of the control bread sample (Figure 4) is characterized by the presence of a single monolithic protein framework, in which starch grains are compactly packed.

The control sample is characterized by the presence of a single swollen structureless elastic jelly. Individual starch grains or the protein matrix are not visualized.

In the crumb samples (a) and (b), the presence of small pores is visualized, bounded by the interstitial walls that make up the spongy skeleton. The interstitial walls consist of a solid mass of protein coagulated during baking, which is interspersed with swollen gelatinized starch grains, which are almost impossible to visually determine, since they are closely adjacent to the mass of coagulated protein with their entire surface, and therefore, there is no sharp, clearly visible border between them. Also, on the surface, there is a developed network of thin protein fibrils. It should be noted that the protein matrix in samples (a), (b), and (c) is more developed, which is due to the intensification of the fermentation process due to the introduction of WWF.

## 4. Conclusions

As a result of the study of the rheological properties of the dough, the quality of bread, and its rheological characteristics, as well as microscopic analysis of the samples, the optimal size of the replacement of refined flour with whole wheat flour from sprouted wheat grain was determined, which was 20%. This percentage of replacement led to better values of the farinograph quality index (200 ± 3 mm).

The bread obtained according to this recipe had a high specific volume (4.21 ± 0.62 mL.g^−1^) and optimal rheological characteristics: total deformation13.7 ± 0.3 mm, plastic4.3 ± 0.3 mm, and elastic9.4 ± 0.3 mm. The study of the microstructure of dough and bread also confirmed the established dependencies.

It is recommended to conduct further studies to study the nutritional value and antioxidant activity of whole wheat flour from sprouted wheat and the dynamics of changes during the baking process, as well as to evaluate the effectiveness of sprouted WWF in various final products of cereals to improve their functionality or increase their nutritional value.

## Figures and Tables

**Table 1:** The formulations of dough.

Ingredients, g	Bread samples			
	Control	a	b	c
Refined wheat flour (RF)	1000	900	800	700
Whole wheat flour from sprouted wheat (WWF)	—	100	200	300
Salt	15	15	15	15
Yeast	20	20	20	20

**Table 2:** Chemical composition of refined wheat flour and whole wheat flour from sprouted and nonsprouted wheat grains.

Indicator name	Refined wheat flour (RF)	Whole grain wheat flour (WGWF)	Whole wheat flour from sprouted wheat (WWF)
Hagberg falling number (FN), sec.	245^1^ ± 10^2^^ab^	310 ± 11^ab^	75 ± 10^ba^
Weighted average particle size, microns	94 ± 15^acb^	168 ± 15^ab^	154 ± 15^bc^
Protein, (% db^3^)	14.3 ± 0.3^dc^	12.5 ± 0.3^d^	13.2 ± 0.3^c^
Gluten (% db)	26.4 ± 3.5^ec^	20.3 ± 2.5^e^	22.6 ± 2.5^c^
Gluten index	68.4 ± 0.2^fg^	62.3 ± 0.2^g^	73.8 ± 0.3^f^
Lipids (% db)	0.3 ± 0.2^ch^	1.8 ± 0.2^h^	1.7 ± 0.2^c^
Starch (% db)	70.6 ± 0.5^fd^	62.4 ± 0.4^d^	41.9 ± 0.3^f^
Moisture (%)	12.9 ± 0.3^c^	13.2 ± 0.4^ac^	13.9 ± 0.3^a^
Ash (% db)	0.8 ± 0.2^f^	2.2 ± 0.2^fc^	2.0 ± 0.2^c^

^1^Means ±  ^2^standard deviation. Means in a row without a common superscript letter differ statistically (*p* < 0.05). ^3^db: dry basis.

**Table 3:** Rheological properties of the obtained dough samples (Farinograph Test).

Quality indicator	Control	a	b	c
Water absorption, %	61^1^ ± 0.9^2^^abc^	59.6 ± 0.4^ab^	58.6 ± 0.6^ac^	56.2 ± 0.4^c^
Dough development time, min	2.5 ± 0.3^dcf^	2.8 ± 0.5^c^	3.3 ± 0.3^d^	4.7 ± 0.3^f^
Dough stability, min	8.2 ± 0.3^fd^	10.8 ± 0.7^d^	11.4 ± 0.5^f^	4.4 ± 0.3^f^
Decrease of consistency after 10 min, E.F.	22 ± 1.5^d^	9 ± 2.5^d^	5 ± 2.6^d^	50 ± 1.9^d^
Decrease of consistency after 12 min, E.F.	31 ± 1.3^c^	16 ± 1.5^c^	16 ± 1.1^d^	99 ± 1.8^d^
Farinograph quality indicator, mm	136 ± 3^acb^	196 ± 5^ab^	200 ± 3^ac^	76 ± 4^cb^

^1^Means ±  ^2^standard deviation. Means in a row without a common superscript letter differ statistically (*p* < 0.05).

**Table 4:** Results of determining the specific volume, deformation, and moisture content of bread.

Samples	Specific volume (mL.g^−1^)	Moisture (%, wb^3^)
Control	3.66^1^ ± 0.09^2^^ab^	40.2 ± 0.5^cd^
a	3.92 ± 0.40^b^	40.8 ± 0.7^d^
b	4.21 ± 0.62^ab^	41.4 ± 0.5^c^
c	3.24 ± 0.09^a^	41.6 ± 0.9^c^

^1^Means ±  ^2^standard deviation. Means in a row without a common superscript letter differ statistically (*p* < 0.05). ^3^wb: wet basis.

## Data Availability

Data available are on request. The data used to support the findings of this study are available from the corresponding author upon request (naumenkonv@susu.ru).

## Abbreviations

RF: Refined wheat flour
WGWF: Whole grain wheat flour
WWF: Whole wheat flour from sprouted wheat.
